# Determinants of non-adherence to disease-modifying therapies in multiple sclerosis: A cross-Canada prospective study

**DOI:** 10.1177/1352458516657440

**Published:** 2016-06-29

**Authors:** Kyla A McKay, Helen Tremlett, Scott B Patten, John D Fisk, Charity Evans, Kirsten Fiest, Trudy Campbell, Ruth Ann Marrie, for the CIHR Team in the Epidemiology and Impact of Comorbidity on Multiple Sclerosis (ECoMS)

**Affiliations:** Division of Neurology, Faculty of Medicine, Djavad Mowafaghian Centre for Brain Health, University of British Columbia, Vancouver, BC, Canada; Division of Neurology, Faculty of Medicine, Djavad Mowafaghian Centre for Brain Health, University of British Columbia, Vancouver, BC, Canada; Departments of Psychiatry and Community Health Sciences, Cumming School of Medicine, University of Calgary, Calgary, AB, Canada; Departments of Psychiatry, Psychology and Neuroscience, Dalhousie University, Halifax, NS, Canada; College of Pharmacy and Nutrition, University of Saskatchewan, Saskatoon, SK, Canada; Departments of Critical Care Medicine & Community Health Sciences, O’Brien Institute for Public Health, and Hotchkiss Brain Institute, University of Calgary, Canada; School of Nursing, Faculty of Health Professions, Dalhousie University, Halifax, NS, Canada; Departments of Internal Medicine and Community Health Sciences, Health Sciences Centre, Max Rady College of Medicine, Rady Faculty of Health Sciences, University of Manitoba, Winnipeg, MB, Canada

**Keywords:** Multiple sclerosis, adherence, immunomodulatory therapy, glatiramer acetate, beta-interferon, health behavior

## Abstract

**Background:**

Poor adherence to the disease-modifying therapies (DMTs) for multiple sclerosis (MS) may attenuate clinical benefit. A better understanding of characteristics associated with non-adherence could improve outcomes.

**Objective:**

To evaluate characteristics associated with non-adherence to injectable DMTs.

**Methods:**

Consecutive patients from four Canadian MS Clinics were assessed at three time points over two years. Clinical and demographic information included self-reported DMT use, missed doses in the previous 30 days, health behaviors, and comorbidities. Non-adherence was defined as <80% of expected doses taken. We employed generalized estimating equations to examine characteristics associated with non-adherence at all time points with findings reported as adjusted odds ratios (OR).

**Results:**

In all, 485 participants reported use of an injectable DMT, of whom 107 (22.1%) were non-adherent over the study period. Non-adherence was associated with a lower Expanded Disability Status Scale score (0–2.5 vs 3.0–5.5, OR: 1.80; 95% confidence interval (CI): 1.06–3.04), disease duration (⩽5 vs <5 years, OR: 2.23; 95% CI: 1.10–4.52), alcohol dependence (OR: 2.14; 95% CI: 1.23–3.75), and self-reported cognitive difficulties, measured by the Health Utilities Index-3 (OR: 1.55; 95% CI: 1.08–2.22).

**Conclusions:**

Nearly one-quarter of participants were non-adherent during the study. Alcohol dependence, perceived cognitive difficulties, longer disease duration, and mild disability status were associated with non-adherence. These characteristics may help healthcare professionals identify patients at greatest risk of poor adherence.

## Introduction

The first disease-modifying therapies (DMTs) for the treatment of relapsing-remitting (RR) multiple sclerosis (MS) were approved in the mid-1990s, and continue to be used as first-line treatments for MS today.^1^ Maintaining adherence to long-term therapies is notoriously challenging.^2^ Estimates of adherence in MS vary widely; a recent review suggested that between 41% and 88% of persons are adherent to the DMTs.^3^ As recognized by the World Health Organization, “Adherence to therapies is a primary determinant of treatment success. Poor adherence attenuates optimum clinical benefits and therefore reduces the overall effectiveness of health systems.”^2^ In MS, non-adherence has been associated with increases in MS-related hospitalizations and relapse rates.^4,5^ To improve adherence, an understanding of potentially modifiable factors that are associated with non-adherence is needed.

Measuring self-reported missed doses is a practical, efficient, and commonly used method in research and clinical practice.^6–8^ Factors associated with missing doses in MS are varied, but include perceived lack of efficacy, adverse drug effects, and simply forgetting to inject.^6–10^ Depression, anxiety, and cognitive difficulties have also been associated with poor drug adherence in MS; however, findings have been inconsistent.^3,7,11–13^ Less is known about the effect of MS symptoms such as pain, fatigue, and other comorbidities. Having to manage complex coexisting conditions is an established determinant of poor adherence,^2^ such that it is conceivable these conditions may influence adherence rates. Adverse health behaviors, such as cigarette smoking and alcohol dependence have not been well-studied in the context of MS and adherence. They may reflect a process of passive coping^14^ which might also lead to poor adherence. If we can establish a demographic pattern of non-adherence, we could potentially develop a more targeted approach to improving adherence.

We estimated adherence rates by accessing self-reported missed doses of the injectable DMTs in a cross-Canada prospective study and examined clinical and demographic patient characteristics potentially associated with non-adherence.

## Methods

### Study population

This DMT adherence study was nested within a broader study of MS patients, details of which have been reported previously.^15^ Briefly, consecutive patients attending a routine visit at one of four MS Clinics in British Columbia, Alberta, Manitoba, and Nova Scotia were recruited between July 2010 and March 2011. Individuals were followed at three time points (“baseline,” year 1, and year 2) which coincided with each participant’s typical annual clinic visit schedule. To maximize response rates, follow-up questionnaires were also offered via telephone, mail, or email for individuals unable to attend their visit. Inclusion criteria for the study were as follows: a neurologist-confirmed diagnosis of definite MS or clinically isolated syndrome (CIS) according to the prevailing criteria;^16–18^ age ⩽18 years; resident in the province where data collection was occurring; and ability and willingness to provide informed consent and to complete the study questionnaires in English. Specifically for this study, participants were included if use of an injectable DMT (interferon beta (IFNβ) or glatiramer acetate) was reported at least once during the study period (at baseline, year 1, or year 2). These injectable drugs were the predominant DMT for MS at the time of data collection, and the questionnaire specifically asked about the number of injections missed in the previous 30 days. Institutional ethics approval was obtained at all sites and participants provided informed consent.

### Clinical and demographic information

Demographic and clinical information were captured from each individual’s medical record using a standardized data abstraction form including sex, date of birth, date of MS symptom onset, clinical course (RR, secondary progressive (SP), primary progressive (PP), and CIS), as per the treating neurologist. Expanded Disability Status Score (EDSS) was captured at baseline and at each follow-up visit (categorized as mild (0–2.5), moderate (3.0–5.5), or severe (6.0+)). Highest education level achieved (categorized as high school or less, any post-secondary or more, and other) and race were also captured at baseline.

Participants completed questionnaires at the baseline, 1-, and 2-year follow-up visits. The questionnaire captured DMT use (by brand name) and number of missed injections in the previous 30 days. Comorbidities were recorded via a validated questionnaire;^19^ the total number of physical comorbidities for each participant was calculated and categorized as 0, 1, or ⩽2. The Hospital Anxiety and Depression Scale (HADS) measured current symptoms of depression and anxiety.^20^ Validated cut-off scores of ⩽8 on the HADS scale were used to define the presence of both.^21^ The Cutting down, Annoyance by criticism, Guilty feeling, Eye-openers (CAGE) questionnaire was used as a screening tool, with a score of ⩽2 out of a possible four suggesting alcohol dependence.^22^ Smoking status was captured as either “current” or “non”-smoker. Fatigue was measured using the Daily Fatigue Impact Scale, an 8-item scale, dichotomized as “no fatigue” (<5) and “any fatigue” (⩽5).^23^ Health-related quality of life (HRQOL) was measured using the Health Utilities Index Mark III version (HUI-3),^24,25^ a 15-item measure that assesses health state with respect to eight single-attribute scores: vision, hearing, speech, mobility, dexterity, emotion, cognition, and pain. These single attribute scores were combined into an overall score which can range from 0 (equivalent to death) to 1 (perfect health), which was categorized as “no to moderate disability” (>0.70) and “severe disability” (⩾0.70).^26^ Individual attribute scales for pain and cognition were also examined separately as both are associated with MS, and with drug adherence in other patient populations.^6,27^ These were categorized as follows: pain that disrupts normal activities (yes vs no; ⩾0.77 vs >0.77) and moderate to severe cognitive difficulties (yes vs no; ⩾0.70 vs >0.70).

### Quantifying adherence

The medication possession ratio (MPR)^3^ was used to estimate adherence, calculated as the number of doses taken divided by the number of expected doses, expressed as a percentage at each study visit. An MPR of ⩽80% was considered adherent, and <80% as non-adherent^8,11,28,29^ (see Table S1, Supplementary Tables for details). This definition was chosen as it is a common binary cut-off in MS DMT adherence literature,^3^ thereby lending itself to comparisons with prior research. Furthermore, non-adherence defined as <80% has been shown to be associated with important clinical outcomes in MS, including increased relapse rates, inpatient visits, and overall medical costs.^4^ As a supplementary analysis, non-adherence was defined as any missed dose during the 30-day period (yes vs no).

### Statistical analysis

Clinical and demographic characteristics of the DMT users were described as frequencies (percentages), mean and standard deviation (SD), or median and interquartile range (IQR). We employed generalized estimating equations (GEE) with an unstructured correlation matrix to examine characteristics associated with non-adherence at all three time points. This approach allowed for the simultaneous analysis of both patient characteristics and adherence measures from all three time points, accounting for correlations between the repeated measures for individuals. If a person was on a DMT at only one of the three visits, only this visit would be included in the analysis; if they were on a DMT at all three visits, all three would be included. Sex, race, and education were captured at baseline only and included as constant variables, while the remaining predictor variables were included as time-varying covariates over the three visits. We employed univariate logistic regression using GEE, followed by a multivariable logistic regression based on the significance (*p* > 0.1) of characteristics from the univariate analysis. To estimate the predictive effect of baseline non-adherence on non-adherence at follow-up (year 1 and 2), we used logistic regression modeling, adjusted for confounders (all measured at baseline). Findings were reported as odds ratios (ORs) with 95% confidence intervals (CI). Analyses were performed using the Statistical Analysis System (SAS) Software Package 9.4 (SAS Institute Inc., Cary, NC).

## Results

Of 1632 patients who visited one of the four MS clinics, 949 consented and participated in the primary study,^15^ of which, 485 reported use of an injectable DMT during the study period and were included in the analyses (Table 1). Nine participants missed their year 1 visit, and 21 missed the year 2 visit, for a retention rate of 95.7%. Females and those of younger age and shorter disease duration were more likely to be on a DMT. The average age of DMT users was 45.5 years, average disease duration was 12.6 years, and most had RRMS (90%) (Table 1).

**Table 1.** Baseline demographic and clinical characteristics of patients who reported taking a disease-modifying drug at some point during the study period (baseline, year 1, or year 2) compared to participants who did not report taking an injectable therapy during the study period.

Baseline characteristics	Exposed to an injectable DMT during study period (*n* = 485)	Unexposed to an injectable DMT during study period (*n* = 464)	*p* value
Sex, *N* (%)			
Female	383 (79.0)	331 (71.3)	0.007^a^
Male	102 (21.0)	133 (38.7)	
Race, *N* (%) (*36 missing*)			
White	427 (94.3)	383 (95.0)	0.615^a^
Non-White	26 (5.7)	20 (5.0)	
Age, mean (SD)	45.5 (10.2)	52.1 (11.4)	<0.0001^b^
Age range (years)	19–71	19–80	
Age of symptom onset, mean (SD)	32.9 (9.1)	33.7 (10.3)	0.240^b^
Disease duration, mean (SD)	12.6 (8.7)	18.4 (9.0)	<0.0001^b^
EDSS, median (IQR)	2.0 (1.5–3.5)	3.5 (2.0–6.0)	<0.0001^c^
Clinical course, *N* (%)			
RRMS	435 (89.7)	252 (54.4)	<0.0001^d^
SPMS	47 (9.7)	146 (31.5)	
PPMS	0 (0.0)	60 (13.0)	
CIS	0 (0.0)	5 (1.1)	
RRMS at onset, but unknown if reached SPMS	3 (0.6)	0 (0.0)	
Education			
High school or less	135 (29.6)	123 (30.8)	0.453^a^
Any post-secondary or more	311 (68.2)	263 (65.8)	
Other	10 (2.2)	14 (3.5)	

DMT: disease-modifying therapy; SD: standard deviation; IQR: interquartile range; EDSS: Expanded Disability Status Score; RRMS: relapsing-remitting multiple sclerosis; SPMS: secondary progressive multiple sclerosis; PPMS: primary progressive multiple sclerosis; CIS: clinically isolated syndrome.

a Pearson’s chi-squared test.

b Student’s *t* test.

c Wilcoxon rank-sum test.

d Fisher’s exact test.

At baseline, 46% (435/949) of the participants were on a first-line DMT, which remained relatively steady over the follow-up period. The frequency of use for each DMT also remained largely stable over the follow-up period although use of the second-line DMTs, fingolimod and natalizumab, increased with time (Supplementary Table S2).

At baseline, 11% (48/426) of participants were non-adherent (MPR: <80%), 13% (50/386) at year 1, and 14% (48/341) at year 2. The denominators reflect the number of responders to the question of missed doses. Non-responders at each time point totaled 9, 13, and 6, respectively (Table 2).

**Table 2.** Frequency of non-adherence, defined as medication possession ratio <80% at baseline, year 1, and year 2 by DMT product.

DMT (route and frequency)	Baseline	Year 1	Year 2
Glatiramer acetate (subcutaneous, daily), *N* (%)	8/131 (6.1)	11/130 (8.5)	7/126 (5.6)
Interferon β-1a (intramuscular, weekly), *N* (%)	10/90 (11.1)	14/76 (18.4)	12/65 (18.5)
Interferon β-1b (subcutaneous, every other day), *N* (%)	12/62 (19.4)	8/51 (15.7)	8/43 (18.6)
Interferon β-1a (subcutaneous, three times per week), *N* (%)	18/143 (12.6)	17/129 (13.2)	21/107 (19.6)

DMT: disease-modifying therapy.

Denominator represents the total number of participants who were on the specified DMT at each visit and who responded to the question “how many doses did you miss in the previous 30 days.” Non-responders to this question at each time point totaled 9, 13, and 6, respectively. Numerator represents the number of people who were not adherent to that DMT. Percentages are shown in parentheses.

During the entire study, 22.1% (107/485) of participants were estimated to be non-adherent at least once. Over half (51%; 255/485) of participants reported missing at least one dose of their DMT in the previous 30 days over the study period.

Findings from the longitudinal, uni- and multivariable analysis are shown in Table 3. After adjusting for potential confounders, alcohol dependence, EDSS, disease duration, DMT product, and perceived cognitive difficulties were associated with non-adherence (Table 3). Those who met criteria for alcohol dependence had more than twice the odds of non-adherence compared to those who did not. Relative to participants with moderate disability (EDSS 3.0–5.5), those with mild disability (EDSS 0–2.5) were more likely to be non-adherent. Longer disease duration (⩽5 vs <5 years) was associated with increased odds of non-adherence. Glatiramer acetate users were more likely to be adherent relative to all three types of IFNβ (Table 3).

**Table 3.** Univariate and multivariable longitudinal analyses of clinical and demographic variables and their association with non-adherence (defined as medication possession ratio <80% in previous 30 days)

Variable	Univariate odds ratio (95% CI)	Multivariable odds ratio (95% CI)
Age (continuous)	0.98 (0.96–1.00)	0.98 (0.96–1.01)
Sex		
Female (reference)	1.00	1.00
Male	1.33 (0.84–2.10)	1.32 (0.84–2.08)
Race		
White (reference)	1.00	
Non-White	1.36 (0.54–3.40)	
Education		
High school or less	1.00	
Post-secondary or higher	1.05 (0.64–1.73)	
Site		
British Columbia (reference)	1.00	
Alberta	0.67 (0.31–1.47)	
Manitoba	0.64 (0.31–1.31)	
Nova Scotia	0.66 (0.41–1.07)	
EDSS		
EDSS mild (0–2.5)	1.76 (1.06–2.92)	1.80 (1.06–3.04)
EDSS moderate (3.0–5.5) (reference)	1.00	1.00
EDSS severe (6.0+)	1.27 (0.64–2.55)	1.30 (0.63–2.66)
Disease course		
Relapsing-remitting (reference)	1.00	
Secondary progressive	0.83 (0.43–1.62)	
Disease duration (years)		
<5 (reference)	1.00	1.00
⩽5	1.78 (0.91–3.38)	2.23 (1.10–4.52)
Disease-modifying therapy (route and frequency)		
Glatiramer acetate (subcutaneous, daily) (reference)	1.00	1.00
Interferon β-1a (intramuscular, weekly)	**3.21 (1.65–6.24)**	**2.83 (1.43–5.62)**
Interferon β-1b (subcutaneous, every other day)	**3.49 (1.60–7.60)**	**3.23 (1.49–6.98)**
Interferon β-1a (subcutaneous, three times per week)	**2.85 (1.51–5.37)**	**2.74 (1.48–5.12)**
Number of physical comorbidities		
0 (reference)	1.00	
1	1.07 (0.72–1.60)	
⩽2	1.10 (0.71–1.70)	
Health Utilities Index (health-related quality of life)		
None to moderate disability (HUI score > 0.70) (reference))	1.00	
Severe disability (HUI score: ⩾0.70)	0.79 (0.55–1.15)	
Health behaviors, mental health, and symptoms of MS		
No alcohol dependence (reference)	1.00	1.00
Alcohol dependence	**2.28 (1.29–4.05)**	**2.14 (1.23–3.75)**
Non-smoker (reference)	1.00	
Current smoker	1.21 (0.70–2.09)	
No depression (reference)	1.00	
Depression	1.42 (0.93–2.17)	
No anxiety (reference)	1.00	
Anxiety	1.10 (0.76–1.59)	
No fatigue (reference)	1.00	
Fatigue	0.93 (0.63–1.38)	
No pain (reference)	1.00	
Pain	1.00 (0.68–1.46)	
None to mild perceived cognitive difficulties (reference)	1.00	1.00
Moderate to severe perceived cognitive difficulties	1.32 (0.94–1.86)	**1.55 (1.08–2.22)**

CI: confidence interval; EDSS: Expanded Disability Status Score; HUI: Health Utilities Index; MS: multiple sclerosis; DMT: disease-modifying therapy.

Variables were measured at baseline, year 1, and year 2 and included in the analysis as time varying, with the exception of sex, race, education, and site which were collected at baseline only.

Odds ratio of >1 indicates a higher odds of non-adherence. Multivariable model was adjusted for age, sex, EDSS, disease duration, DMT product, alcohol dependence, and perceived cognitive difficulties.

The odds of missing any doses were assessed in the supplementary analysis (Supplementary Tables S3). When adjusting for potential confounders in the multivariable model (Supplementary Table S3), similar factors emerged as significant as in the primary adjusted analysis, including alcohol dependence, cognition, and DMT product. However, with this alternative method of assessing adherence, the odds of non-adherence were greater for the glatiramer acetate users (subcutaneous, daily) relative to the IFNβ-1a users (weekly intramuscular or subcutaneous three times per week). In addition, younger age and the presence of multiple physical comorbidities (⩽2 relative to none) were associated with a higher odds of non-adherence.

Finally, previous non-adherence (determined at baseline using the MPR) was associated with over four times the odds of future non-adherence at year 1 or 2 (OR: 4.42; 95% CI: 2.23–8.75), adjusting for sex, and baseline age, disease duration, EDSS, and alcohol dependence.

## Discussion

Over one in five participants in this large, cross-Canada cohort reported missing more than 20% of their doses and less than half were fully adherent in the last 30 days. Our study identified specific characteristics that influenced the likelihood of adherence, including some modifiable attributes. These characteristics may put an individual at risk of non-adherence or be useful markers of future non-adherence for the treating health professional. Of the patient-related characteristics explored, previous non-adherence, alcohol dependence, perceived cognitive difficulties, longer disease duration, and mild disability (EDSS), emerged as factors associated with non-adherence.

Rates of adherence were stable over time, and within the range of estimates from previous studies;^3^ however, variability in the definition of adherence makes direct comparisons between studies challenging.^3^ Authors who also employed a MPR cut-off of 80% reported estimates between 70% and 85%,^8,28^ consistent with our overall adherence rate of 78%. Baseline non-adherence was the most significant predictor of future non-adherence in our study, suggesting that poor adherence is an enduring pattern of behavior for some individuals. This may also serve as a useful early marker of poor future adherence. These results are in concordance with another MS study^7^ and in chronic diseases in general,^27^ and may provide an opportunity for early identification of individuals who may benefit from additional, ongoing support.

Alcohol dependence was associated with twice the risk of non-adherence. We are aware of only one prior study examining this risk factor in MS, which found that alcohol was the strongest predictor of missed doses among people with MS living in Tasmania, Australia.^7^ These findings may be of particular concern given the broader negative effects of alcohol dependence,^15^ and given that individuals with MS have been reported as having high rates of alcohol dependence.^30^ Smoking has not been extensively studied in relation to adherence; a single study that had examined the association in MS found no relationship, similar to our findings.^7^

There was no association between mental health (anxiety or depression, measured by the HADS) and missed drug doses. This is consistent with another study using similar methods.^7^ Other studies that used different measures for both mental health (e.g. Beck Depression Inventory) and adherence have shown an association between depression and missed doses in MS although neither considered the effect of alcohol use on adherence.^10,11^ In fact, one excluded patients who abused alcohol,^11^ which could account for the conflicting results, as there is a complex and bidirectional relationship between alcohol dependence and depression.^15^ However, depression can increase the likelihood of treatment discontinuation in MS.^31^ Thus, further study of this issue is warranted; particularly given the high prevalence of mental health disorders in those with MS.^32^

The risk of poor adherence increased with increasing disease duration, consistent with a previous study.^6^ However, the odds of non-adherence modestly decreased with age, as observed in other disease states.^2^ Interestingly, persons with mild disability (EDSS) were less likely to be adherent. These individuals may perceive themselves as having less serious disease and hence, are less motivated to take their medication. They may benefit from an open dialogue as to the rationale for taking drug and expectations related to drug treatment.

Overall, HRQOL and pain, as measured by the HUI-3, were not associated with adherence. However, self-reported cognitive difficulties as reported on the HUI-3 were consistently an important determinant of non-adherence in our study and in some other chronic diseases.^27^ We found one other MS study in which the relationship between cognition and adherence was assessed.^11^ Using an extensive battery of cognitive tests in 55 MS individuals, who were primarily taking glatiramer acetate, associations with adherence were found.^11^ The HUI cognition scale, as used in our study, provides a more pragmatic option, being of lower burden to patients and feasible to implement in routine clinical practice. It specifically addresses forgetfulness, which might be the key element related to missed doses or poor adherence.^6^

In previous studies, persons with multiple comorbid diseases have expressed challenges with adhering to their medication regimens, especially when on multiple medications.^33^ Our findings suggest that MS patients with multiple physical comorbidities had increased odds of missing at least one dose of DMT; however, no relationship was found between the total number of physical comorbidities as categorized by 0, 1, or ⩽2 and adherence, when adherence was defined by the MPR.

Interestingly, the relationship between the different DMT products and adherence varied considerably, depending on the definition of adherence. Glatiramer acetate had the most frequent (daily) dosing schedule of the injectable DMTs. It was associated with better adherence relative to IFNβ, when adherence was defined as ⩽80% of expected doses taken, but not when using “any missed dose.” Previous research has suggested that people do not adhere well to glatiramer acetate relative to IFNβ.^3^ Together, these highlight the substantial impact that the definition and method for measuring adherence may have on findings. Clarity on this issue is important for future studies examining the clinical implications of poor adherence and when comparing study findings.

Our study included a large multi-site sample, had a high retention, and recruited from at least two sites which served as the only source of MS care in their regions, all suggesting that this was a representative sample of clinic-attending MS patients. A potential limitation was the use of self-report, such that our non-adherence rates might be considered conservative. However, this study used a specific recall period of 30 days, and collected the information via survey to reduce desirability bias as recommended elsewhere.^34^

Reporting bias is a possibility; those that are more likely to report adverse health behaviors, such as alcohol dependence, may also be more likely to report their missed doses. There is also a possibility of recall bias, in that some participants, especially those with cognitive difficulties, may not remember how many doses they missed in the last 30 days.

Since the implementation of this study, several oral and other parenterally administered therapies have become available. A single study has reported better adherence among fingolimod users relative to users of the injectable therapies,^35^ but determinants of non-adherence to the oral therapies have not yet been established. It is conceivable that the characteristics recognized in this study may also contribute to missed doses of oral therapies. Future studies should address this important question.

Adherence to medication is central in the treatment of chronic disease to help derive the maximum possible clinical benefit. In the wider medical literature, poor adherence has been linked to worsening morbidity, death, and increased healthcare costs.^36^ In MS, poor adherence has been associated with an increased risk of MS relapse, and MS-related hospitalization.^4^ In this study, nearly one-quarter of participants reported poor adherence to their DMT at least once during the study period, and over half were not fully adherent. Healthcare professionals should be aware of the greater potential for poor adherence among patients with low levels of disability, longer disease duration, and a history of poor adherence. Alcohol dependence and perceived cognitive difficulties were also important markers of non-adherence. Improving adherence is an ongoing process that involves patients, healthcare providers, and health systems. Enhancing communication between health professionals (including neurologists, general practitioners, nurses and pharmacists), patients, and their families; implementing educational interventions for those at risk; and addressing modifiable risk factors such as alcohol dependence could effectively improve health outcomes in individuals with MS and ultimately reduce costs to health systems.^36^

## Supplementary Material

Supplementary material

## References

[1] Filippini G Del Giovane C Vacchi L Immunomodulators and immunosuppressants for multiple sclerosis: A network meta-analysis. Cochrane Libr 2013; 6: 1–134. 10.1002/14651858.CD008933.pub2 PMC11627144 23744561

[2] World Health Organization. Adherence to long-term therapies: evidence for action, 2003 Available from: http://www.who.int/chp/knowledge/publications/adherence_report_fin.pdf?ua=1

[3] Menzin J Caon C Nichols C Narrative review of the literature on adherence to disease-modifying therapies among patients with multiple sclerosis. J Manag Care Pharm 2013; 19: 24–40. 10.18553/jmcp.2013.19.s1.S24 PMC10437533 23383731

[4] Tan H Cai Q Stephenson JJ Impact of adherence to disease-modifying therapies on clinical and economic outcomes among patients with multiple sclerosis. Adv Ther 2011; 28: 51–61. 21153000 10.1007/s12325-010-0093-7

[5] Steinberg SC Faris RJ Chang CF Impact of adherence to interferons in the treatment of multiple sclerosis: A non-experimental, retrospective, cohort study. Clin Drug Investig 2010; 30: 89–100. 10.2165/11533330-000000000-00000 20067327

[6] Devonshire V Lapierre Y Macdonell R The Global Adherence Project (GAP): A multicenter observational study on adherence to disease-modifying therapies in patients with relapsing-remitting multiple sclerosis. Eur J Neurol 2011; 18: 69–77. 20561039 10.1111/j.1468-1331.2010.03110.x

[7] Tremlett H Van der Mei I Pittas F Adherence to the immunomodulatory drugs for multiple sclerosis: Contrasting factors affect stopping drug and missing doses. Pharmacoepidemiol Drug Saf 2008; 17: 565–576. 18395883 10.1002/pds.1593

[8] Siegel SD Turner AP Haselkorn JK Adherence to disease-modifying therapies in multiple sclerosis: Does caregiver social support matter? Rehabil Psychol 2008; 53: 73–79.

[9] Río J Porcel J Téllez N Factors related with treatment adherence to interferon beta and glatiramer acetate therapy in multiple sclerosis. Mult Scler J 2005; 11: 306–309. 10.1191/1352458505ms1173oa 15957512

[10] Treadaway K Cutter G Salter A Factors that influence adherence with disease-modifying therapy in MS. J Neurol 2009; 256: 568–576. 19444532 10.1007/s00415-009-0096-y

[11] Bruce JM Hancock LM Arnett P Treatment adherence in multiple sclerosis: Association with emotional status, personality, and cognition. J Behav Med 2010; 33: 219–227. 20127401 10.1007/s10865-010-9247-y

[12] Turner AP Williams RM Sloan AP Injection anxiety remains a long-term barrier to medication adherence in multiple sclerosis. Rehabil Psychol 2009; 54: 116–121. 19618711 10.1037/a0014460

[13] Mohr DC Boudewyn AC Likosky W Injectable medication for the treatment of multiple sclerosis: The influence of self-efficacy expectations and injection anxiety on adherence and ability to self-inject. Ann Behav Med 2001; 23: 125–132. 11394554 10.1207/S15324796ABM2302_7

[14] Carver C Scheier M Weintraub J Assessing coping strategies: A theoretically based approach. J Pers Soc Psychol 1989; 56: 267–283. 2926629 10.1037//0022-3514.56.2.267

[15] McKay KA Tremlett H Fisk JD Adverse health behaviours are associated with depression and anxiety in multiple sclerosis: A prospective multisite study. Mult Scler J 2016; 22: 685–693. 10.1177/1352458515599073 PMC4819567 26245214

[16] Poser CM Paty DW Scheinberg L New diagnostic criteria for multiple sclerosis: Guidelines for research protocols. Ann Neurol 1983; 13: 227–231. 6847134 10.1002/ana.410130302

[17] McDonald WI Compston A Edan G Recommended diagnostic criteria for multiple sclerosis: Guidelines from the International Panel on the diagnosis of multiple sclerosis. Ann Neurol 2001; 50: 121–127. 11456302 10.1002/ana.1032

[18] Polman CH Reingold SC Edan G Diagnostic criteria for multiple sclerosis: 2005 revisions to the “McDonald Criteria.” Ann Neurol 2005; 58: 840–846. 16283615 10.1002/ana.20703

[19] Horton M Rudick RA Hara-Cleaver C Validation of a self-report comorbidity questionnaire for multiple sclerosis. Neuroepidemiology 2010; 35: 83–90. 20551692 10.1159/000311013

[20] Zigmond A Snaith R The hospital anxiety and depression scale. Acta Psychiatr Scand 1983; 67: 361–370. 6880820 10.1111/j.1600-0447.1983.tb09716.x

[21] Honarmand K Feinstein A Validation of the Hospital Anxiety and Depression Scale for use with multiple sclerosis patients. Mult Scler 2009; 15: 1518–1524. 19965520 10.1177/1352458509347150

[22] Ewing JA Detecting alcoholism: The CAGE questionnaire. J Am Med Assoc 1984; 252: 1905–1907. 10.1001/jama.252.14.1905 6471323

[23] Fisk JD Doble SE Construction and validation of a fatigue impact scale for daily administration (D-FIS). Qual Life Res 2002; 11: 263–272. 12074263 10.1023/a:1015295106602

[24] Horsman J Furlong W Feeny D The Health Utilities Index (HUI^®^): Concepts, measurement properties and applications. Health Qual Life Outcomes 2003; 1: 54–67. 14613568 10.1186/1477-7525-1-54 PMC293474

[25] Fisk JD Brown MG Sketris IS A comparison of health utility measures for the evaluation of multiple sclerosis treatments. J Neurol Neurosurg Psychiatry 2005; 76: 58–63. 15607996 10.1136/jnnp.2003.017897 PMC1739294

[26] Feng Y Bernier J Mcintosh C Validation of disability categories derived from Health Utilities Index mark 3 scores. Heal Reports 2009; 20: 43–50. 19728585

[27] Dunbar-Jacob J Mortimer-Stephens MK Treatment adherence in chronic disease. J Clin Epidemiol 2001; 54: S57–S60. 11750211 10.1016/s0895-4356(01)00457-7

[28] Halpern R Agarwal S Dembek C Comparison of adherence and persistence among multiple sclerosis patients treated with disease-modifying therapies: A retrospective administrative claims analysis. Patient Prefer Adherence 2011; 5: 73–84. 21423591 10.2147/PPA.S15702 PMC3058604

[29] Turner AP Kivlahan DR Sloan AP Predicting ongoing adherence to disease modifying therapies in multiple sclerosis: Utility of the health beliefs model. Mult Scler 2007; 13: 1146–1152. 17967842 10.1177/1352458507078911

[30] Marrie RA Reingold S Cohen J The incidence and prevalence of psychiatric disorders in multiple sclerosis: A systematic review. Mult Scler 2015; 21: 305–317. 25583845 10.1177/1352458514564487 PMC4429164

[31] Mohr DC Goodkin DE Likosky W Therapeutic expectations of patients with multiple sclerosis upon initiating interferon beta-1b: Relationship to adherence to treatment. Mult Scler 1996; 2: 222–226. 9050360 10.1177/135245859600200502

[32] Marrie RA Fisk JD Tremlett H Differences in the burden of psychiatric comorbidity in MS vs the general population. Neurology 2015; 85: 1972–1979. 26519542 10.1212/WNL.0000000000002174 PMC4664123

[33] Bayliss EA Steiner JF Fernald DH Descriptions of barriers to self-care by persons with comorbid chronic diseases. Ann Fam Med 2003; 1: 15–21. 15043175 10.1370/afm.4 PMC1466563

[34] Stirratt MJ Dunbar-Jacob J Crane HM Self-report measures of medication adherence behavior: Recommendations on optimal use. Transl Behav Med 2015; 5: 470–482. 26622919 10.1007/s13142-015-0315-2 PMC4656225

[35] Bergvall N Petrilla A Karkare SU Persistence with and adherence to fingolimod compared with other disease-modifying therapies for the treatment of multiple sclerosis: A retrospective US claims database analysis. J Med Econ 2014; 17: 696–707. 25019581 10.3111/13696998.2014.940422

[36] Osterberg L Blaschke T Adherence to medication. N Engl J Med 2005; 353: 487–497. 16079372 10.1056/NEJMra050100

