# Supplementary material for: Determinants of non-adherence to disease-modifying therapies in multiple sclerosis: A cross-Canada prospective study
Source: Mult Scler. 2016 Jun 29;23(4):588–96. doi: 10.1177/1352458516657440 (PMC5407504; doi:10.1177/1352458516657440)
Supplement: Supplementary material [file MSJ657440_supplementary_tables.pdf]

Tables
Determinants of non-adherence to disease-modifying therapies in multiple sclerosis

Supplementary Tables

		**Number of doses required in order to fulfill the definition of:**	
**Disease-modifying therapy (route and frequency)**	Expected number of doses in 30 days	'Adherent' (i.e, ≥ 80% of expected doses taken)	'Non-adherent' (i.e, < 80% of expected doses taken)
**Interferon β-1a (intramuscular, weekly)**	4	4	< 4
**Interferon β-1b (subcutaneous, every other day)**	15	≥ 12	< 12
**Interferon β-1a (subcutaneous, three times per week)**	12	≥ 10	< 10
**Glatiramer acetate (subcutaneous, daily)**	30	≥ 24	< 24

Table S1. Definitions of adherent and non-adherent based on the medication possession ratio of 80% for the four disease-modifying therapy (DMT) types.
Key: Each number shown in the table represents the number of doses of injectable DMTs during a 30-day period. The 'expected number' for each DMT were derived from the relevant product monograph and represent the full, licensed dosing schedule for each drug.

Disease-modifying therapy (route and frequency)	Baseline	Year One	Year Two
**None‡**	47 (9.7)	62 (13.1)	78 (17.0)
**Interferon β-1a (intramuscular, weekly)**	93 (19.2)	79 (16.6)	66 (14.4)
**Interferon β-1b (subcutaneous, every other day)**	63 (13.0)	52 (11.0)	43 (9.4)
**Interferon β-1a (subcutaneous, three times per week)**	146 (30.2)	132 (27.8)	111 (24.1)
**Glatiramer acetate (subcutaneous, daily)**	133 (27.5)	136 (28.6)	127 (27.6)
**Natalizumab (intravenous, every 28 days)**	1 (0.2)	8 (1.7)	14 (3.0)
**Fingolimod (oral, daily)**	0 (0.0)	2 (0.4)	14 (3.0)
**Other**	2 (0.2)	4 (0.8)	7 (1.5)

Table S2. Disease-modifying therapy (DMT) use at baseline, year one, and year two among study participants.
'Other' includes clinical trials participants, mitoxantrone users, or unknown.
‡all individuals were exposed to an injectable DMT at some point during follow-up, but not necessarily at every time point

Tables

Determinants of non-adherence to disease-modifying therapies in multiple sclerosis

**Variable**	**Univariate odds ratio (95%CI)**	**Multivariable odds ratio (95 % CI)**
**Age (continuous)**	0.99 (0.98 – 1.01)	**0.98 (0.96 – 0.99)**
Sex		
**Female (reference)**	1.00	1.00
**Male**	**1.53 (1.06 – 2.21)**	1.31 (0.89 – 1.92)
Race		
**White (reference)**	1.00	
**Non-White**	1.01(0.49 – 2.10)	
Education		
**High school or less**	1.00	
**Post-secondary or higher**	1.09 (0.76 – 1.54)	
Site		
**British Columbia (reference)**	1.00	
**Alberta**	0.78 (0.46 – 1.32)	
**Manitoba**	0.98 (0.58 – 1.67)	
**Nova Scotia**	0.73 (0.52 – 1.04)	
EDSS		
**EDSS mild (0-2.5)**	1.04 (0.78 – 1.39)	
**EDSS moderate (3.0-5.5) (reference)**	1.00	
**EDSS severe (6.0+)**	0.82 (0.53 – 1.29)	
Disease Course		
**Relapsing-remitting (reference)**	1.00	
**Secondary Progressive**	0.76 (0.49 – 1.17)	
Disease Duration		
**< 5 years (reference)**	1.00	
**≥ 5 years**	1.35 (0.92 – 1.98)	
Disease-modifying therapy (route and frequency)		
**Glatiramer acetate (subcutaneous, daily) (reference)**	1.00	1.00
**Interferon β-1a (intramuscular, weekly)**	**0.23 (0.14 – 0.39)**	**0.22 (0.13 – 0.37)**
**Interferon β-1b (subcutaneous, every other day)**	1.21 (0.75 – 1.95)	1.26 (0.78 – 2.03)
**Interferon β-1a (subcutaneous, three times per week)**	**0.59 (0.75 – 0.85)**	**0.60 (0.41 – 0.86)**
Number of physical comorbidities		

**0 (reference)**	1.00	1.00
**1**	1.14 (0.83 – 1.57)	1.33 (0.96 – 1.84)
**≥ 2**	**1.39 (1.00 – 1.94)**	**1.54 (1.07 – 2.21)**
Health Utilities Index (Health-related quality of life)		
**None to moderate disability (HUI score > 0.70) [reference])**	1.00	
**Severe disability (HUI score ≤ 0.70)**	**1.32 (1.02 – 1.72)**	
Health behaviours, mental health, and symptoms of MS		
**No alcohol dependence (reference)**	1.00	1.00
**Alcohol dependence**	**2.39 (1.51 – 3.78)**	**2.61 (1.57 – 4.34)**
**Non-smoker (reference)**	1.00	
**Current Smoker**	1.13 (0.77 – 1.66)	
**No depression (reference)**	1.00	
**Depression**	1.09 (0.81 – 1.48)	
**No anxiety (reference)**	1.00	
**Anxiety**	1.14 (0.88 – 1.46)	
**No fatigue (reference)**	1.00	
**Fatigue**	1.23 (0.93 – 1.64)	
**No pain (reference)**	1.00	
**Pain**	1.13 (0.88 – 1.44)	
**None to mild perceived cognitive difficulties (reference)**	1.00	1.00
**Moderate to severe perceived cognitive difficulties**	**1.44 (1.13 – 1.84)**	**1.38 (1.05 – 1.80)**

Table S3. Univariate and multivariable longitudinal analyses of clinical and demographic variables and their association with non-adherence (missed ≥ 1 dose in the previous 30 days; ‘yes’ vs ‘no’). Variables were measured at baseline, year one, and year two and included in the analysis as time-varying, with the exception of sex, race, education, and site which were collected at baseline only.

Odds ratio of > 1 indicates a higher odds of non-adherence. Multivariable model was adjusted for age, sex, DMT product, physical comorbidity count, alcohol dependence, and perceived cognitive difficulties.
